# IgG4-related sialadenitis complicated with type III mixed cryoglobulinemia: A case report

**DOI:** 10.1097/MD.0000000000016571

**Published:** 2019-08-02

**Authors:** Rui-Yu Zhang, Zhi-Rui Zhao, Xiao-Yi Xu, Li-Jun Sun, Hong-Rui Dong, Hong-Liang Rui, Guo-Qin Wang, Hong Cheng, Yi-Pu Chen

**Affiliations:** Division of Nephrology, Beijing Anzhen Hospital, Capital Medical University, Beijing, P. R. China.

**Keywords:** IgG4-related sialadenitis, cryoglobulinemia, IgG4^**+**^ plasmocytes, storiform fibrosis

## Abstract

**Rationale:**

IgG4-related disease (IgG4-RD) is a systemic autoimmune disease and mixed cryoglobulinemia may be caused by autoimmune diseases. However, so far only 1 case of IgG4-RD complicated with mixed cryoglobulinemia is reported. Our case further confirms the close relationship between these 2 diseases.

**Patient concerns:**

A 55-year-old female was admitted because of dry mouth and teeth falling off.

**Diagnoses:**

The patient was diagnosed as IgG4-related sialadenitis (IgG4-RS) complicated with type III mixed cryoglobulinemia. IgG4-RS was confirmed by elevated serum IgG4 levels and diffuse IgG4^**+**^ plasmocyte infiltration and storiform fibrosis in the interstitium of labial gland. Type III mixed cryoglobulinemia was confirmed by positive serum cryoglobulins and no monoclonal immunoglobulin in serum and urine.

**Interventions and Outcomes:**

After treatment with prednisone and cyclophosphamide, serum cryoglobulins rapidly turned negative with the remission of IgG4-RS.

**Lessons:**

Type III mixed cryoglobulinemia can be caused by IgG4-RS, and the underlying mechanisms need to be further explored.

## 1 Introduction

IgG4-related disease (IgG4-RD) is a systemic autoimmune disease. According to reports, more than forty different organs have been involved by IgG4-RD.^[1]^ The top 5 frequently involved organs are as follows: submandibular glands 28%, lymph nodes 27%, orbit 22%, pancreas 19% and retroperitoneum 18%.^[1]^ The typical histopathologic features in involved organs are significant IgG4 positive plasmacytic infiltration, storiform fibrosis, and obliterative phlebitis.^[2]^

Cryoglobulins are immunoglobulins that precipitate at temperatures less than 37°C and redissolve after rewarming. Cryoglobulinemia refers to the presence of cryoglobulins in serum. Cryoglobulins are classified into the following 3 types: type I consists of monoclonal immunoglobulin; type II is composed of monoclonal IgM with rheumatoid factor activity and polyclonal IgG; ty**p**e III is constituted by polyclonal IgM with rheumatoid factor activity and polyclonal IgG. Type II and III are also referred to as mixed cryoglobulins.^[3]^

Autoimmune disease is an important cause of mixed cryoglobulinemia, but so far only 1 case of cryoglobulinemia caused by IgG4-RD has been reported by Kimaya et al.^[4]^ Now we report another case here. The case study was approved by the Ethics Review Committee of Beijing Anzhen Hospital (approval number: 2019011X), and informed written consent was obtained from the patient for publication of this case report.

## 2 Case presentation

A 55-year-old female has felt dry mouth for several months, even needing water to help swallow food sometimes. Most of her teeth have gradually taken off over the past few years. She did not feel obvious dry eyes. One month ago urinalysis found protein (+) in her urine. She never had skin purpura, ulcer, and arthralgia. Physical examination showed normal blood pressure (109/65mmHg), only 5 teeth remaining in mouth, no palpable swelling or masses in salivary glands, and no edema in lower extremities.

**Figure 1:**
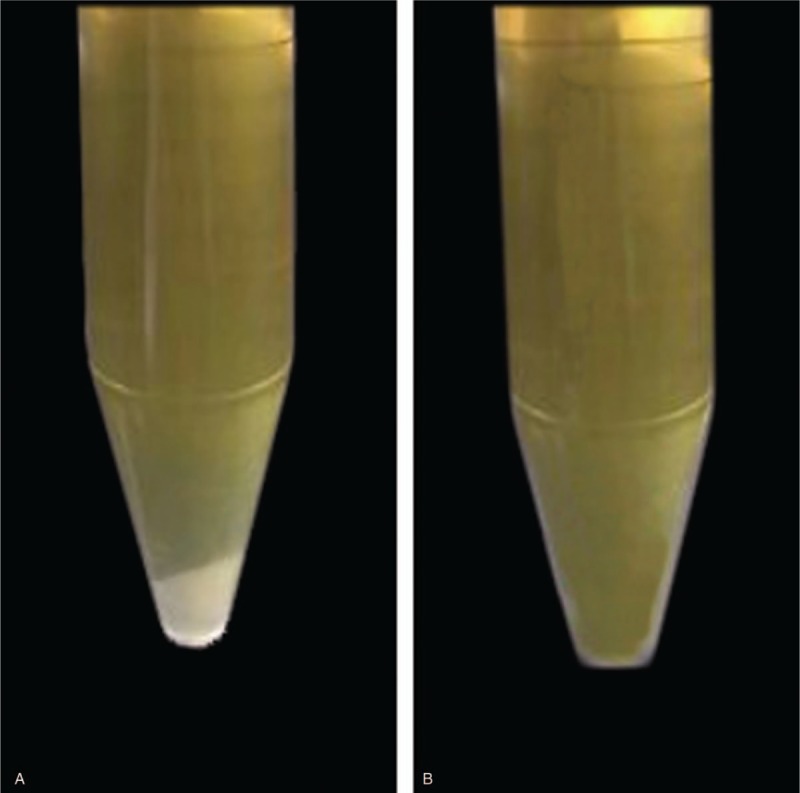
Serum cryoglobulin qualitative test. (A)Serum cryoprecipitates appeared after 7 days of incubation at 4°C. (B) Serum cryoprecipitates redissolved after rewarming at 37°C.

Laboratory tests revealed mild proteinuria (0.3 g/d) with normal urinary sediment. Serum creatinine (50 μmol/L) and blood urea nitrogen (6.5mmol/L) were both normal. Serum globulin level was markedly elevated (55.9 g/L). The levels of serum IgG (43.48 g/L) and IgG4 (29.80 g/L) were also markedly elevated .The levels of serum IgA, IgM, IgG1, IgG2, and IgG3 were all normal. The levels of serum C3 (0.52 g/L) and C4 (0.04 g/L) were significantly reduced. Rheumatoid factor level was significantly increased (147.6 IU/ml). Serum cryoglobulin test was positive (Fig. 1). Anti-dsDNA, anti-SSA, anti-SSB, and anti-Ro 52 antibodies were all negative with weakly positive antinuclear antibody. Both nucleic acid and immunological tests for HCV, HIV, and HBV were negative. Serum protein electrophoresis, serum, and urine immunofixation electrophoresis all revealed no monoclonal immunoglobulin.

Saliva flow rate was abnormal (<0.2 mL/15 min). Radionuclide examination of salivary glands showed mild impairment of the uptake and excretion functions in the left parotid gland, and moderate impairment in the bilateral submandibular glands. Schirmer test and tear breakup time test of both eyes were positive.

**Figure 2:**
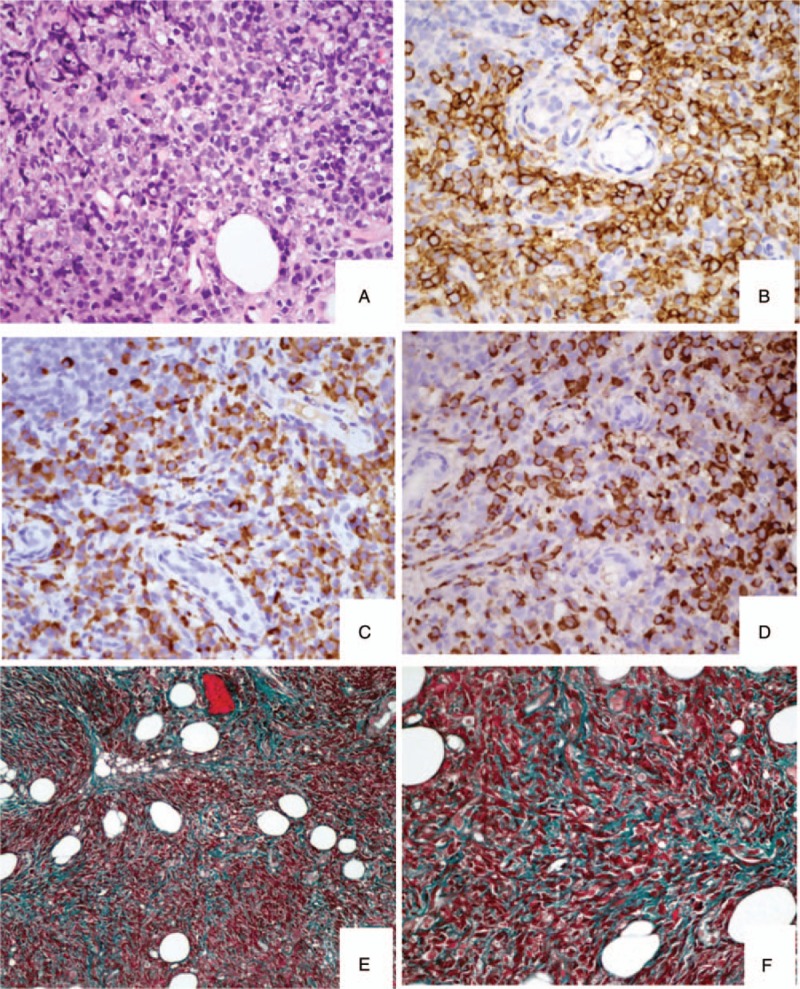
Pathological findings of the labial gland biopsy tissue. **(A)** Diffuse infiltration of mononuclear cells and plasmocytes in interstitium (HE, × 400). **(B, C, D)** Infiltration of abundant plasmocytes (CD 138^+^ cells), IgG^+^ cells and IgG4^+^ cells in interstitium (immunostaining, × 400). **(E, F)** Storiform fibrosis in interstitium (Masson, × 200 and × 400, respectively).

Pathological examination of labial gland biopsy tissue revealed that diffuse inflammatory cells infiltration around ducts and atrophic acinus. The infiltrating inflammatory cells were predominantly IgG4^**+**^ plasmocytes (>150/HPF) and the ratio of IgG4^**+**^/IgG^**+**^ plasmocytes was 50-70%. Storiform fibrosis was observed in interstitium (Fig. 2). The pathological diagnosis was IgG4-related sialadenitis (IgG4-RS).

**Figure 3:**
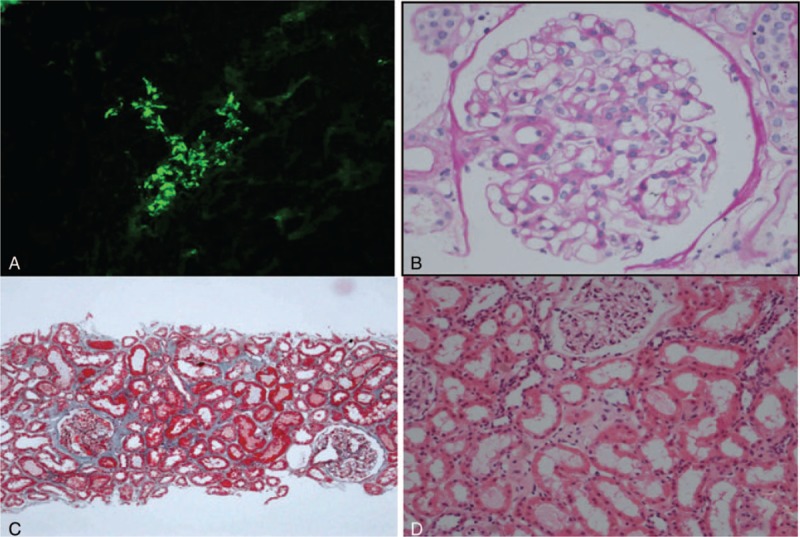
Pathological findings of the kidney biopsy tissue. **(A)** IgM granular deposits in the mesangium (Immunofluorescence, × 200). **(B)** Mild proliferation of mesangial cells (PAS, × 400). **(C)** No storiform fibrosis in interstitium (Masson, × 100). **(D)** A few mononuclear cells in interstitium (HE, × 400).

Renal biopsy was also performed. Immunofluorescence examination showed IgM (2+), IgG, IgA and C3 (1+), C1q and FRA (−) in mesangium. Immunohistochemistry staining showed no IgG4^**+**^ plasmocytes in interstitium. Light microscopy displayed mild proliferation of mesangial cells and slight increase of mesangial matrix. No thrombus-like protein was found within glomerular capillary lumena. No storiform fibrosis was observed in interstitium (Fig. 3). Electron microscopy revealed electron dense deposits in mesangium. No deposits with fibrillary or microtubular substructure were observed in glomeruli. The pathological diagnosis was IgM nephropathy.

The patient was treated with prednisone (initial dose 30 mg/d) and oral cyclophosphamide (cumulant 8.5 g). After 9 months of treatment, patient's dry mouth symptoms improved significantly. Serum globulin and IgG levels both recovered to normal. Serum IgG4 level significantly dropped to near normal (3.28 g/L). Serum C3 and C4 levels both elevated to normal. Serum cryoglobulin test became negative. Urinalysis returned to normal.

## 3 Discussion

The comprehensive diagnostic criteria for IgG4-RD established by Japan College of Rheumatology in 2011 are the following:

1. clinical examination shows characteristic diffuse/localized swelling or masses in single or multiple organs;
2. hematological examination shows elevated serum IgG4 concentrations (≥135 mg/dL);
3. histopathological examination shows:
  (a) marked lymphocyte and plasmocyte infiltration and fibrosis;
  (b) infiltration of IgG4^**+**^ plasmocytes: ratio of IgG4^**+**^**/** IgG^**+**^ cells >40% and >10 IgG4^**+**^ plasmocytes/HPF.^[5]^

Our patient meets the above criteria (2) and (3). Although no palpable swelling or mass of the salivary glands was found at the time of hospitalization, she felt dry mouth and the uptake and excretion functions of salivary glands were impaired. We consider that no enlargement of salivary glands might be related to the chronic transformation of salivary gland lesions and obvious interstitial fibrosis. International consensus guidance statement published in 2015 pointed out that the histopathologic and immunohistochemistry features that support the diagnosis of IgG4-RD, in the proper clinical setting, can be viewed as diagnostic.^[6]^ Therefore our patient can be affirmatively diagnosed as IgG4-RS.

Positive serum rheumatoid factor and decreased C3 and C4 levels are important clues to suggest mixed cryoglobulinemia.^[3,7]^ Based on this, we performed serum cryoglobulin test for the patient and the result was positive. So, she also suffered from cryoglobulinemia even without the clinical manifestations of vasculitis. Her cryoglobulinemia belongs to type III mixed cryoglobulinemia because no monoclonal immunoglobulin can be found in her serum and urine. Mixed cryoglobulinemia is often caused by infections such as HCV, HIV, or HBV infection, or autoimmune diseases such as Sjogren's syndrome, systemic lupus erythematosus or rheumatoid arthritis.^[3,7]^ Our patient has no evidence of infection and the autoimmune diseases mentioned above. So, we think that her type III cryoglobulinemia may be caused by IgG4-RS. After treatment with prednisone and cyclophosphamide, her serum cryoglobulins rapidly turned negative with the remission of IgG4-RS, which also suggests that the 2 diseases are closely related.

The mechanism by which IgG4-RD causes cryoglobulinemia remains unclear. In 2016, Kimaya et al^[4]^ hypothesized that dysfunction of B lymphocytes may be an underlying pathogenic mechanism of both IgG4-RD and cryoglobulinemia, in which B cell-activating factor (BAFF) may play an important role. It has been found that serum BAFF levels in patients with IgG4-RD were significantly elevated, while glucocorticoid therapy could dramatically reduce the levels of serum BAFF and IgG4.^[8]^ It has also observed in a patient with Sjogren's syndrome complicated with cryoglobulinemic vasculitis and elevated serum BAFF levels that serum cryoglobulins continuously turned negative after treatment with belimumab (a BAFF blocker).^[9]^ So, the hypothesis put forward by Kimaya et al^[4]^ deserves attention and should be further studied and verified in the future.

Finally, the kidney disease in this patient is not caused by IgG4-RD or cryoglobulinemia. The major kidney lesion of IgG4-RD is tubulointerstitial nephritis with marked IgG4+ plasmacytic infiltration and storiform fibrosis in interstitium.^[1,10]^ The renal involvement of cryoglobulinemic vasculitis manifests chiefly as membrano-proliferative glomerulonephritis with thrombus-like cryoglobulin deposits within capillary lumina.^[7,11]^ Therefore, the patient's kidney disease, IgM nephropathy, is an independent disease.

In summary, we report here a case of IgG4-RD complicated with type III mixed cryoglobulinemia. The underlying mechanisms leading to the coexistence of these 2 diseases need to be further explored.

## Author contributions

**Data curation:** Ruiyu Zhang, Zhirui Zhao, Xiaoyi Xu.

**Formal analysis:** Guoqin Wang, Hong Cheng.

**Resources:** Lijun Sun, Hongrui Dong, Hongliang Rui.

**Supervision:** Guoqin Wang, Hong Cheng, Yipu Chen.

**Validation:** Hong Cheng, Yipu Chen.

**Visualization:** Lijun Sun, Yipu Chen.

**Writing – original draft:** Ruiyu Zhang.

**Writing – review & editing:** Yipu Chen.

## References

[1] Vasaitis L IgG4-related disease. A relatively new concept for clinicians. Eur J Intern Med 2016;27:1–9. 26481243 10.1016/j.ejim.2015.09.022

[2] Wallace ZS Deshpande V Mattoo H IgG4-related disease: clinical and laboratory features in one hundred twenty-five patients. Arthritis Rhenmatol 2015;67:2466–75. 10.1002/art.39205 PMC4621270 25988916

[3] Kolopp-Sarda MN Miossec P Cryoglobulins: an update on detection, mechanisms and clinical contribution. Autoimmun Rev 2018;17:457–64. 29526627 10.1016/j.autrev.2017.11.035

[4] Kamiya M Shane PY Soejima M IgG4-related sialoadenitis with a skin lesion and multiple mononeuropathies suggesting coexistent cryoglobulinemic vasculitis. Intern Med 2016;55:1355–61. 27181547 10.2169/internalmedicine.55.5332

[5] Umehara H Okazaki K Masaki Y Comprehensive diagnostic criteria for IgG4-related disease (IgG4-RD), 2011. Mod Rheumatol 2012;22:21–30. 22218969 10.1007/s10165-011-0571-z

[6] Khosroshahi A Wallace ZS Crowe JL International consensus guidance statement on the management and treatment of IgG4-related disease. Arthritis Rheumatol 2015;67:1688–99. 25809420 10.1002/art.39132

[7] Rui HL Cheng H Wang GQ Clinical and pathological analysis of 30 cases of cryoglobulinemia-associated glomerulonephritis. Chin J Prec Intern Med 2018;38:553–8.

[8] Kiyama K Kawabata D Hosono Y Serum BAFF and APRIL levels in patients with IgG4-related disease and their clinical significance. Arthritis Res Ther 2012;14:R86. 22531553 10.1186/ar3810 PMC3446460

[9] De Vita S Quartuccio L Salvin S Sequential therapy with belimumab followed by rituximab in Sjögren's syndrome associated with B-cell lymphoproliferation and overexpression of BAFF: evidence for long-term efficacy. Clin Exp Rheumatol 2014;32:490–4. 24802131

[10] Salvadori M Tsalouchos A Immunoglobulin G4-related kidney diseases: an updated review. World J Nephrol 2018;7:29–40. 29359118 10.5527/wjn.v7.i1.29 PMC5760510

[11] Fogo AB Lusco MA Najafian B AJKD Atlas of renal pathology: cryoglobulinemic glomerulonephritis. Am J Kidney Dis 2016;67:e5–7. 26802335 10.1053/j.ajkd.2015.12.007

